# Improving physical health for psychiatric patients detained in a low secure forensic psychiatric unit in the United Kingdom

**DOI:** 10.1192/bjo.2021.490

**Published:** 2021-06-18

**Authors:** Cosmina Cross

**Affiliations:** Southern Health NHS Trust

## Abstract

**Aims:**

This project aimed to improve physical health and to tackle obesity in patients detained in a low secure forensic psychiatric unit.

**Background:**

People suffering from severe and enduring mental health problems have a life expectancy of 15-20 years less than the general population. The main cause of death is cardiovascular disease due to lifestyle factors, such as smoking, substance misuse and obesity.

Physical health problems such as metabolic syndrome, diabetes and heart disease have a knock on effect on motivation, self-esteem and concordance with treatment.

Over 60% of the general population and up to 80% of patients detained in forensic psychiatric units in the UK are classed as overweight or obese, with serious consequences to physical health.

Southfield Low Secure Unit is a 28 bed unit. Most patients suffer from treatment resistant schizophrenia and are prescribed high doses of antipsychotic medication, some up to 250% of the maximum recommended dose.

**Method:**

Baseline data were collected using Body Mass Index (BMI) and Simple Physical Activity Questionnaire (SPAQ). Following the initial data collection, patients were involved in focus groups, community meetings and a monthly physical health action group. There was input from the care team including psychology, occupational therapy, nursing, catering and security. New activities have been made available such as “physical health and mental health education group”, “rambling group”, “gym sessions”, “patient focus groups” and “walking group”.

**Result:**

This project has been running for 9 months and is ongoing.

There has been a modest change in the BMI – initial results ranging from BMI 23.6–42.8kg/m2. Of the initial cohort (n = 14), there has been weight loss (n = 3), weight gain (n = 3) and no change (n = 8).

The initial SPAQ results showed that on average patients spend 19.8 hours per day either in bed or doing sedentary activities and only 1.68 hours per day walking or doing physical activities. This pattern is being reassessed.

The qualitative data from patient focus groups shows increased interest in activities, motivation and desire to contribute to the project.

**Conclusion:**

The preliminary results show an increase in patient motivation and engagement with available activities. There have also been patient-led challenges which were well received. Patients feel positive about the programme and valued for their input. Further support is required to maintain progress.

